# Spontaneous Aortocaval Fistula with Abdominal Aortic Aneurysm 5 Years after Aortic Valve Replacement Complicated by Aortic Dissection

**DOI:** 10.1055/s-0039-1683882

**Published:** 2019-04-24

**Authors:** Sobhi Mleyhi, Mohamed Messai, Mohamed Ben Hammamia, Skander Ben Omrane

**Affiliations:** 1 Department of Cardiovascular and Thoracic Surgery, Medical University of Tunis, Rabta Hospital, Tunis, Tunisia

**Keywords:** aortic valve replacement, aortocaval fistula, endovascular treatment

## Abstract

Iatrogenic aortic dissection after cardiac surgery is a rare and serious complication, the management of which involves many therapeutic modalities and whose prognosis is associated with high rate of morbidity and mortality. The authors report the case of a 61-year-old man presented with aortocaval fistula and aortic dissection 5 years after aortic valve replacement.

A 61-year-old man with medical history of hypertension, smoking, and aortic valve replacement 5 years ago with a size 23 mechanical St. Jude aortic prosthesis presented with a 4-day history of lower abdominal pain and hemodynamic compromise. Abdominal examination revealed a tender, pulsatile aorta, and blood tests revealed acute renal failure. An urgent computed tomographic scan was performed. It revealed a dissection of the ascending aorta ( Fig. 1 ) with a large abdominal aortic aneurysm ( Fig. 2 ) associated with an aortocaval fistula ( Fig. 3 ). An endovascular treatment was proposed given the anticoagulated state (international normalized ratio at 2.75), but the patient died after a rapid deterioration of his hemodynamic status. We suppose that the aortic dissection, which was probably due to the past cardiac surgery, was the primary cause of the dissecting aneurysm of the abdominal aorta, which was subsequently complicated by aortocaval fistula. 1 In such a case, endovascular treatment has its place, given the severity of the patient's hemodynamic status and the surgical difficulty 2 due to the primary pathology and the anticoagulated state.

**Fig. 1:**
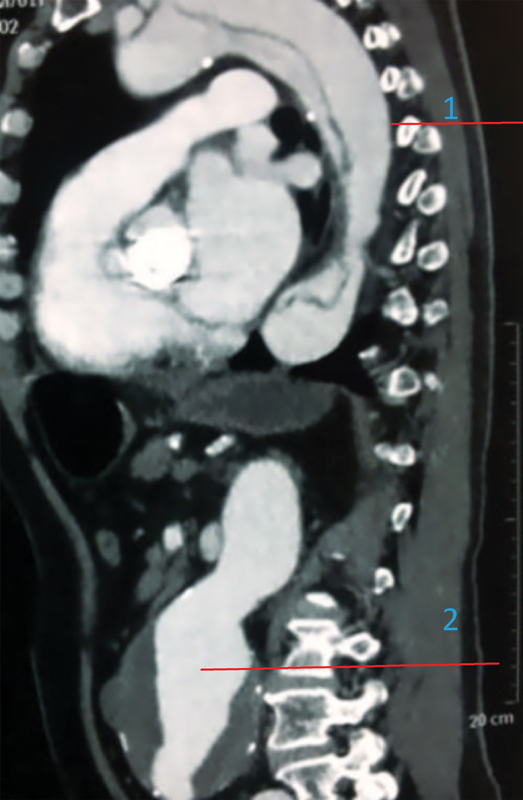
Sagittal computed tomography (CT) scan that documents aortic dissection (Type A) and aortic aneurysm. 1, aortic dissection with the true and the false channel; 2, aortic aneurysm.

**Fig. 2:**
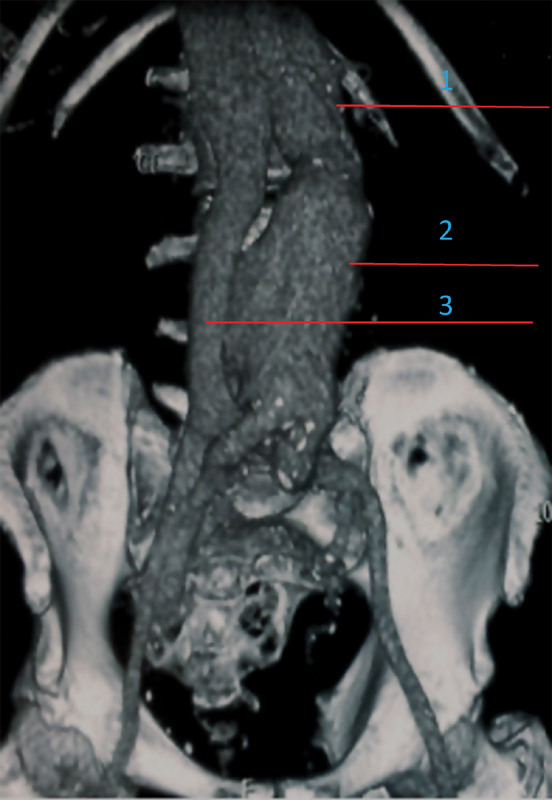
Coronal reconstruction showing aneurysm of the aorta with early opacification of the inferior vena cava secondary to the aortocaval fistula. 1, nonaneurysmal aorta; 2, aneurysmal aorta; 3, inferior vena cava.

**Fig. 3:**
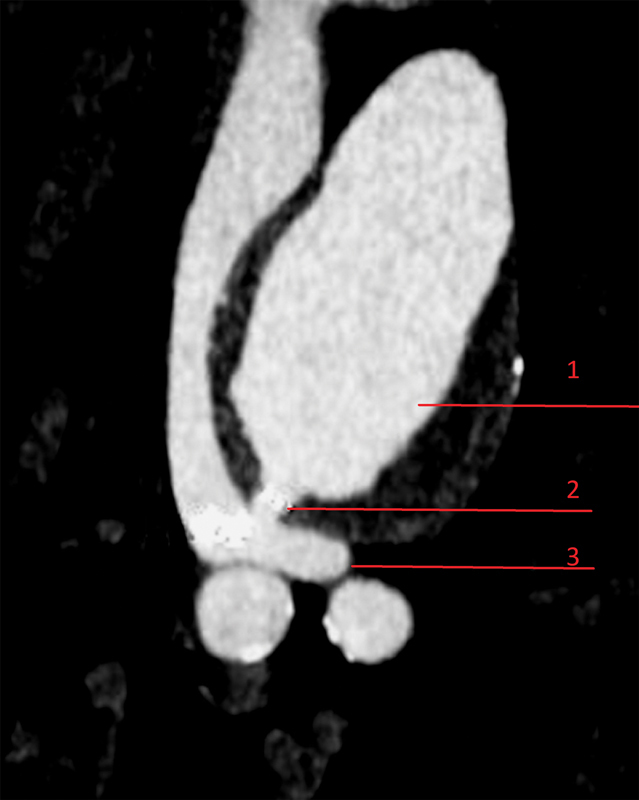
Axial section of the aortocaval fistula. 1, aorta; 2, fistula; 3, inferior vena cava.
